# Design and In Vitro Evaluation of Cyclodextrin-Functionalized Albumin Nanoparticles for Intranasal Carbamazepine Brain Delivery

**DOI:** 10.3390/pharmaceutics18030331

**Published:** 2026-03-06

**Authors:** Hanan Mohammad, Maher Darwish, Mária Budai-Szűcs, Maryana Salamah, Rita Ambrus, György Tibor Balogh, Gábor Katona, Ildikó Csóka

**Affiliations:** 1 Institute of Pharmaceutical Technology and Regulatory Affairs, Faculty of Pharmacy, University of Szeged, H-6720 Szeged, Hungary; hanan.adnan.mohammad@szte.hu (H.M.); budai-szucs.maria@szte.hu (M.B.-S.); salamah.maryana@szte.hu (M.S.); ambrus.rita@szte.hu (R.A.); csoka.ildiko@szte.hu (I.C.); 2 Department of Optics and Quantum Electronics, University of Szeged, Dóm Sq. 9, H-6720 Szeged, Hungary; darwish_maher@ymail.com; 3 Department of Pharmaceutical Chemistry and Drug Control, Faculty of Pharmacy, Wadi International University, Homs, Syria; 4 Department of Pharmaceutical Chemistry, Semmelweis University, Hőgyes Endre Str. 9, H-1092 Budapest, Hungary; balogh.gyorgy.tibor@semmelweis.hu; 5 Center for Pharmacology and Drug Research & Development, Semmelweis University, Üllői Str. 26, H-1085 Budapest, Hungary; 6 Department of Chemical and Environmental Process Engineering, Budapest University of Technology and Economics, Műegyetem Quay 3, H-1111 Budapest, Hungary

**Keywords:** carbamazepine, bovine serum albumin, nanoparticle, cyclodextrin, functionalization

## Abstract

**Background/Objectives**: Poor aqueous solubility and limited nasal permeability remain key challenges in the intranasal delivery of carbamazepine. In this study, biocompatible bovine serum albumin nanoparticles functionalized with sulfobutyl-β-cyclodextrin (SβCD-BSA NPs), comprising individually cytocompatible components with confirmed physical interactions), were formulated for intranasal delivery of carbamazepine (CBZ). **Methods**: The ethanolic desolvation method was utilised for the preparation of the nanoparticles, with the functional moiety incorporated during nanoparticle preparation. The effects of different molar ratios of SβCD-BSA and different ethanol volume ratios were studied. For crosslinking, 1-ethyl-3-(3-dimethylaminopropyl) carbodiimide hydrochloride (EDC), a non-toxic crosslinker, was utilised. To determine the role of the SβCD, two preparation samples were formulated, with and without SβCD. **Results**: The formulation without SβCD incorporation had a mean particle size of 125 ± 0.64 nm, polydispersity index (PDI) of 0.34, encapsulation efficiency (EE%) of 61.5 ± 1.40%, and drug-loading ratio (DL%) of 31.9 ± 1.50%. Conversely, the SβCD-functionalized formulation showed a mean particle size of 128 ± 2.12 nm, PDI of 0.21 ± 0.03, EE of 64.6 ± 0.35%, and DL of 34.28 ± 1.60%. Statistical analysis revealed that the incorporation of SβCD resulted in a statistically significant increase in both DL% and EE% (*p* < 0.05). Conversely, the observed differences in particle size and PDI were not statistically significant (*p* > 0.05). This addition provides precise context regarding the comparability of the formulations while highlighting SβCD’s functional benefits in solubility and permeation. The interaction between CBZ and SβCD-BSA was confirmed using Fourier-transform infrared spectroscopy. Lastly, the prepared formulations were characterised by their physicochemical attributes and in vitro biopharmaceutical studies. It was discovered that SβCD plays a dual role, enhancing the solubility of CBZ in one scenario while promoting its nasal permeation, suggesting its potential use in epilepsy treatment. **Conclusions**: These findings highlight the potential of SβCD-BSA NPs as a versatile pharmaceutics platform for the intranasal delivery of poorly soluble CNS drugs.

## 1. Introduction

Epilepsy is one of the most common neurological syndromes, affecting over 50 million people globally, according to the WHO statistics in 2024. This disorder occurs across all age groups, prompting ongoing efforts to develop more effective treatments [1,2,3]. Among the available therapeutic options, there are 30 antiepileptic drugs (AEDs), more than two-thirds of which were introduced to the market after carbamazepine (CBZ) [4]. CBZ is also commonly used to treat trigeminal neuralgia and manic depression. Furthermore, the global market for CBZ is projected to expand significantly, as evidenced by recent market reports forecasting a steady Compound Annual Growth Rate (CAGR) from 2025 to 2030 (Figure S1) [5]. CBZ is a narrow therapeutic index drug with a therapeutic plasma range of 4–12 µg/mL and an oral bioavailability of 75–85% despite extensive hepatic metabolism by CYP3A4; chronic therapy requires gradual dose titration because of autoinduction, which shortens the elimination half-life from about 40 to 20 h over 3–4 weeks and increases clearance up to two-fold, leading to fluctuating plasma levels (C_max_/C_min_ ≈ 2–3). According to the Tegretol^®^ prescribing information, epilepsy treatment is typically initiated at 100–200 mg once or twice daily and titrated to a maintenance dose of 400 mg two to three times daily (maximum 1600–2000 mg/day), with a time to peak plasma concentration (T_max_) of 4–5 h after oral administration [6].

Despite its clinical efficacy, CBZ is limited by poor aqueous solubility (200 µg/mL), fluctuating plasma concentrations due to autoinduction, and variable absorption requiring dose titration. These pharmacokinetic challenges result in suboptimal therapeutic effectiveness during dose adjustment periods. [7,8]. To overcome these drawbacks, intranasal drug delivery can be a promising strategy that circumvents first-pass metabolism, improves patient adherence, offers a rapid onset of action, and facilitates direct delivery from the nasal cavity to the brain [9,10,11]. Such a route is envisioned as an adjunct to, rather than a full replacement for, conventional oral CBZ therapy, aiming to provide rapid-onset nose-to-brain delivery of low doses that help achieve or maintain therapeutic CNS exposure during dose-adjustment periods or acute seizure episodes. Also, the route poses its own challenges, including the limited solubility and high molecular weight of many active pharmaceutical ingredients (APIs) [12], as well as local mucosal irritation and impaired mucociliary clearance, which can hinder drug absorption [13,14].

Nanotechnology has been successfully employed to address these limitations. By precisely regulating the particle size, surface charge, and shape of nanoparticles, more effective delivery and pharmacokinetics have been achieved [15]. For instance, particles in the 20–200 nm size range are best suited for intranasal delivery [16], where they exploit the natural pore structure of nasal mucus and are taken up by cells via clathrin- and caveolae-mediated endocytosis [17]. Furthermore, this size range is best suited to permeate the blood–brain barrier (BBB) and to bypass rapid clearance by the reticuloendothelial system (RES), thereby allowing for a longer circulation period [18,19]. However, nanoparticles below 5 nm are rapidly cleared by renal filtration [20]. Positively charged nanoparticles can enhance interactions with the negatively charged endothelial cells of the BBB [21,22]. However, high concentrations of cationic particles can lead to toxicity [23]. Conversely, neutral and negatively charged nanoparticles, particularly at lower concentrations, exhibit lower toxicity while still ensuring similar circulation times [24,25].

Despite CBZ’s efficacy, poor solubility, first-pass metabolism, and nasal permeation challenges limit its intranasal potential (Table 1). Nanotechnology addresses these via optimised size (20–200 nm), charge, and functionalization (e.g., CDs for solubility/permeation).

Bovine serum albumin (BSA) has recently attracted attention as a biocompatible, non-immunogenic carrier that can cross the BBB, making it an attractive candidate for intranasal delivery to the central nervous system (CNS) [30]. Unfortunately, BSA, like other proteins, encounters obstacles to mucosal bypass, and its absorption decreases significantly with increasing molecular size [31,32]. Furthermore, conventional BSA nanoparticles often exhibit low encapsulation efficiency (EE%) and may aggregate when particle sizes exceed 100 nm [33]. To overcome these challenges, we propose a functionalization strategy that uses cyclodextrins (CDs) conjugated to BSA, as CDs have been used to increase drug solubility, improve drug absorption, and enhance targeting to specific brain regions when incorporated into an intranasal formulation [34,35]. CDs interact with functional groups on amino acids to facilitate the systemic delivery of peptides and proteins [36], but their hydrophilicity and high molecular weight typically preclude their direct neuronal delivery after crossing the BBB [37,38].

Although some studies indicate the formation of a CBZ–cyclodextrin inclusion complex for intranasal administration, cyclodextrins alone are limited by several issues. These include local toxicity, evident from nasal morphological changes, alterations in ciliary beat rate, the release of marker drugs, erythrocyte haemolysis, and cytotoxicity in vitro [31]. Thus, the use of sulfobutylether-β-cyclodextrin (SβCD) and hydroxypropyl-β-cyclodextrin (HβCD) blended with a biocompatible carrier such as BSA provides an effective strategy to overcome these constraints and to secure greater safety and performance. Based on this, the present work was conducted to develop cyclodextrin-functionalized BSA nanoparticles for the intranasal delivery of CBZ, with SβCD ultimately selected as the optimal functional moiety following a DOE-based screening. The nanoparticles were synthesised using the desolvation method and crosslinked with the nontoxic 1-ethyl-3-(3-dimethylaminopropyl) carbodiimide hydrochloride (EDC), then thoroughly characterised and evaluated in vitro. To the best of our knowledge, this is the first study reporting the rational design of SβCD-functionalized BSA nanoparticles to simultaneously address CBZ’s solubility, colloidal stability, and nasal permeation limitations.

## 2. Materials and Methods

### 2.1. Chemicals

BSA (lyophilised powder, purity ≥ 97%), ethanol 96% *v*/*v* (EtOH) and CBZ were purchased from Merck Ltd. (Budapest, Hungary). HβCD, SβCD, bicinchoninic acid (BCA) and EDC were obtained from Sigma-Aldrich Co. Ltd. (Budapest, Hungary). Purified water was prepared using a gradient water purification system (Millipore Milli-Q^®^, Merck Ltd., Budapest, Hungary). Simulated nasal electrolyte solution (SNES) was prepared by dissolving 2.98 g/L of potassium chloride (KCl), 8.77 g/L of sodium chloride (NaCl), and 0.59 g/L of anhydrous calcium chloride (CaCl_2_) from Merck Ltd. in purified water (pH 5.6). Dipotassium hydrogen phosphate, phosphoric acid, and potassium dihydrogen phosphate were obtained from Merck Ltd. Phosphate-buffered solution (PBS) was prepared according to the European Pharmacopoeia 5.0. Cellulose acetate membrane (0.45 μm pore size, >1000 kDa MWCO, Spectra/Por 4 dialysis membrane (12–14 kDa MWCO).

### 2.2. Design of Experiment (DOE) Screening Study

Based on our review of the literature, we identified the factors most commonly studied in similar formulations. To determine which of these variables has the greatest impact, we conducted a screening study using a regular two-level fractional factorial design (2^^ (6−2)^), suitable for efficient six-factor screening. The independent variables and their levels, as predetermined from the literature survey, are summarised in Table 2.

The screening study was based on analysing the effects of these factors on major response variables, including mean particle size, PDI, zeta potential, and nanoparticle yield. Design-Expert^®^ version 13 was used to generate regular fractional factorial design (16 factorial runs plus 2 centre points; total of 18 runs) to systematically assess the main effects and low-order interactions (Table S1). The resulting response surface models (expressed in actual factors) obtained from ANOVA are reported in Table S2.

### 2.3. Preparation of Albumin Nanoparticles and Drug Incorporation

**Figure 1:**
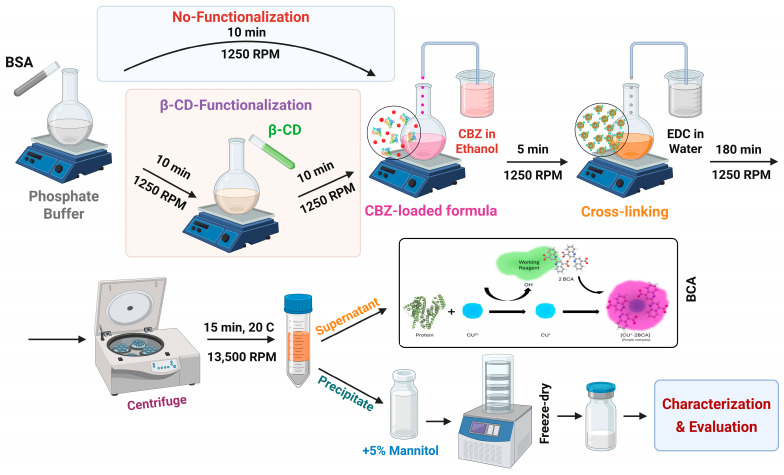
Preparation and purification of the nanoparticles using the desolvation method. Created in BioRender. Maher, H. (2026) https://BioRender.com/wld6x6t.

BSA nanoparticles were synthesised and in situ functionalized using the desolvation method [39], as depicted in Figure 1 and outlined in Table 2. Initially, BSA aqueous solutions were prepared in phosphate buffer at varying pH levels. Subsequently, the βCD derivative was added, and the mixture was stirred for 10 min. EtOH was then added dropwise at 1 mL/min, and the mixture was stirred continuously at 1250 rpm. Following desolvation, the nanoparticle suspension was crosslinked with an aqueous EDC solution for 3 h at 1250 rpm. The βCD-functionalized BSA nanoparticles (HβCD-BSA and SβCD-BSA) were purified through three cycles of ultracentrifugation, each at 13,500 rpm for 30 min at 20 °C. After each centrifugation, the resultant nanoparticles were redispersed in water to restore the original volume. To enhance stability during lyophilization, a 5% *w*/*v* mannitol solution was added as a cryoprotectant. The nanoparticle suspension was then freeze-dried at −40 °C, 0.013 mbar using a Scanvac CoolSafe 100-9 Pro freeze dryer (LaboGene ApS, Lynge, Denmark).

Following completion of the screening process, the integration of CBZ with nanoparticles was carried out to obtain carbamazepine-loaded BSA nanoparticles (CBZ@BSA), carbamazepine-loaded HβCD-BSA nanoparticles (CBZ@HβCD-BSA), and carbamazepine-loaded SβCD-BSA nanoparticles (CBZ@SβCD-BSA). Specifically, CBZ was dissolved in EtOH at a concentration of 3 mg/mL. This solution was added dropwise to the BSA solution to confirm the formulation and enhance CBZ loading.

### 2.4. Determination of Nanoparticle Yield

The efficiency of BSA nanoparticle production was assessed by measuring the amount of unaggregated free BSA remaining in the supernatant after centrifugation, which was not included in the nanoparticle formulation. This evaluation was performed immediately after nanoparticle preparation using the BCA assay kit (Merck Ltd., Budapest, Hungary) [40]. UV–Vis spectra of BCA assay samples were recorded at 562 nm using a Jasco V-730 UV–Vis spectrophotometer (ABL&E-JASCO Ltd., Budapest, Hungary). The nanoparticle yield was then calculated using the following equation:

(1) $$\mathrm{Yield}\;\left(\%\right)=\left(\frac{\mathrm{Total}\;\mathrm{BSA}-\mathrm{Free}\;\mathrm{BSA}\;\mathrm{in}\;\mathrm{supernatant}}{\mathrm{Total}\;\mathrm{BSA}}\right)\times 100$$

### 2.5. Determination of CBZ Content

For the quantification of CBZ in our assessments, the HPLC method developed by Mowafy et al. was used, employing an Agilent Infinity 1260 HPLC system (Agilent Technologies, Santa Clara, CA, USA) equipped with a Kinetex EVO^®^ C18 column (150 mm, 4.6 µm). The mobile phase consisted of methanol and water at a 50:50 ratio, run at a flow rate of 1 mL/min, with detection at λmax of 285 nm [41]. A calibration series was prepared within a linear range of 0.5–40 µg/mL, with a determination coefficient (R^2^) of 0.9988. For any samples with concentrations outside the linear range, appropriate dilutions or concentration (via drying and reconstitution) were performed to ensure precise quantification within the established validated limits.

### 2.6. Colloidal and Morphological Characterisation

Average hydrodynamic diameter (Z-average), PDI, and zeta potential (ζ-potential) were determined in folded-capillary cells using the Zetasizer Nano ZS90 apparatus (Malvern Instruments, Malvern, Worcestershire, UK). Each measurement was performed in triplicate, and data were presented as means ± SD. Additionally, morphological analysis was performed using scanning electron microscopy (SEM; Hitachi S4700, Hitachi Scientific Ltd., Tokyo, Japan). The freeze-dried nanoparticle samples were mounted on aluminium stubs, sputter-coated with gold–palladium, and imaged at an accelerating voltage of 10.0 kV.

### 2.7. Drug Loading (DL%) and Encapsulation Efficiency (EE%)

CBZ content within the nanoparticle formulation was calculated and expressed as a percentage of the total nanoparticle mass. A precise measured amount of the formulation was suspended in 1 mL of water and centrifuged (13,500 rpm, 20 °C for 30 min, in triplicate). The precipitate was transferred to a 10 mL flask, and the volume was made up with acetonitrile (ACN) while stirring for 10 min to assist in drug release. The mixture was centrifuged again, and the supernatant was collected for CBZ quantification using HPLC. Finally, the CBZ content was expressed as a percentage of the total mass of the drug-loaded nanoparticles [42]. EE% was also calculated. The amount of drug was divided by the total amount of CBZ used in the preparation. The equations for calculating DL% and EE% are as follows:

(2) $$\mathrm{DL}\;\left(\%\right)\;=\;\frac{\mathrm{Weight}\;\mathrm{of}\;\mathrm{the}\;\mathrm{drug}\;\mathrm{in}\;\mathrm{NPs}}{\mathrm{Weight}\;\mathrm{of}\;\mathrm{the}\;\mathrm{NPs}}\times 100$$

(3) $$\mathrm{EE}\;\left(\%\right)=\frac{\mathrm{Weight}\;\mathrm{of}\;\mathrm{the}\;\mathrm{drug}\;\mathrm{in}\;\mathrm{NPs}}{\mathrm{Weight}\;\mathrm{of}\;\mathrm{the}\;\mathrm{feeding}\;\mathrm{drug}}\times 100$$

### 2.8. FTIR Measurement

FTIR spectra were recorded on an AVATAR330 FT-IR spectrometer (Thermo Nicolet, Unicam Hungary Ltd., Budapest, Hungary) with a deuterated triglycine sulphate detector, in the spectral range 400–4000 cm^−1^. A 10 mg sample of the freeze-dried formulation was homogenised with 0.15 g of potassium bromide (KBr) in a mortar, then pressed into a 13 mm diameter pellet under a compression force of 10 kN using a Specac^®^ hydraulic press (Specac Inc., Orpington, UK). The spectral resolution was set to 2 cm^−1^, and 128 scans were performed to improve the signal-to-noise ratio. The background spectrum recorded for a pure KBr pellet was subtracted from each spectrum.

### 2.9. In Vitro Assessments

#### 2.9.1. Mucoadhesion Study

For the mucoadhesion study, a TA-XT Plus Texture Analyser (Metron Kft, Budapest, Hungary) with a 5 kg load cell and a 1 cm diameter cylindrical probe was used. A total of 20 mg of the sample was placed on a filter paper fixed to the cylinder probe. The probe was then pressed onto a filter paper pretreated with 50 µL of an 8% *w*/*w* mucin dispersion, simulating the mucosal membrane. The mucin dispersion was prepared in SNES. After a 2500 mN preload for 3 min, the cylinder probe was moved upwards to detach the sample from the artificial mucosa. The maximum adhesive force (detachment force) was recorded, and the adhesive work (A, mN·mm) was calculated from the area under the “force versus distance” curve. A 0.5% *w*/*v* aqueous solution of sodium hyaluronate (NaHa) was used as a reference. Five parallel measurements were performed at 35 °C, and the results were presented as means ± SD.

#### 2.9.2. In Vitro Nasal Diffusion Study

The permeability study was conducted using a modified Side-Bi-Side^®^ horizontal permeation instrument with a cellulose membrane impregnated with isopropyl myristate between the donor and acceptor chambers [43]. The donor compartment contained 8 mL of SNES (pH 5.6) mixed with 1 mL of the redispersed formulation, while the acceptor chamber was filled with 9 mL of PBS (pH 7.4). CBZ has been reported to be chemically stable in simulated nasal media and phosphate buffer pH 7.4 over diffusion and release time frames comparable to those applied here, as demonstrated in previous intranasal CBZ nanoparticulate studies [19].

The test was performed at 32 ± 0.5 °C using a ThermoHaake C10-P5 heating circulator (Sigma–Aldrich Co., Ltd., Budapest, Hungary), under constant stirring at 100 rpm. At scheduled intervals of 30, 60, 120, 180, and 360 min, 100 µL aliquots were withdrawn from the acceptor chamber for HPLC analysis and replaced with the same volume of fresh SNES. The apparent permeability (P_app_) of CBZ across the nasal mucosa at 360 min was quantified using the following equation [44]:

(4) $$P_{app}=\;\frac{{\Delta C}_{A}\times V_{A}}{A\;\times C_{D}\times \;\Delta t}$$

where ΔC_A_ is the concentration differential of CBZ in the acceptor chamber after the experiment, V_A_ is the volume of the acceptor chamber (20 mL), A is the permeability test surface area (2.01 cm^2^), C_D_ is the initial concentration of CBZ in the donor compartment at time point zero, and t is the incubation time (s).

This isopropyl myristate-impregnated cellulose membrane model (0.45 µm) was previously developed and validated by our group for intranasal formulations, showing a strong in vitro–in vivo correlation for nose-to-brain delivery (r ≈ 0.9957, *p* < 0.01) in meloxicam-loaded polymeric micelles [45].

Accordingly, it was selected here as a reproducible, high-throughput barrier for comparative diffusion screening between CBZ formulations. To maintain sink conditions throughout the experiment, the acceptor compartment volume and CBZ loading were selected such that the maximum achievable CBZ concentration in the receptor phase remained well below its solubility in PBS (pH 7.4), and each sampled volume was immediately replaced with fresh buffer.

#### 2.9.3. In Vitro Release Studies

The in vitro release of CBZ from different formulations was evaluated using the dialysis bag diffusion method. Briefly, 1 mL of each formulation, including free CBZ solution, CBZ@BSA, and CBZ@SβCD-BSA, was transferred to dialysis bags. The bags were immersed in 50 mL of freshly prepared SNES (pH 5.6), which served as the release medium. The system was maintained at 32 ± 0.5 °C under constant stirring at 50 rpm. At predetermined intervals (1, 3, 5, 10, 15, 30, and 60 min), 0.5 mL aliquots of the release medium were taken and replaced with an equal volume of fresh medium to maintain sink conditions. The concentration of CBZ released was quantified using the validated HPLC method described earlier. Each measurement was performed in triplicate (*n* = 3), and the results were expressed as means ± SD. The cumulative percentage of drug released over time was then plotted to assess the release kinetics. The same procedure was repeated with PBS (pH 7.4) as the release medium; however, in this medium the study was extended to 360 min to capture the full release profile in a physiological buffer system.

#### 2.9.4. Parallel Artificial Membrane Permeability Assay (PAMPA)

A PAMPA method was used to evaluate the penetration of CBZ across the BBB. Briefly, the sandwich system assessed CBZ’s ability to diffuse from an aqueous solution into a lipid phase and then back into another aqueous solution. To prepare the membrane, 5 µL of lipid solution (8 mg of brain polar lipid extract (porcine) dissolved in 280 μL hexane and 120 μL dodecane) was used. The acceptor plate (Multiscreen Acceptor Plate, MSSACCEPTOR; Millipore, Merck Ltd., Budapest, Hungary) was filled with 300 µL of pH 7.4 PBS. Then, 150 µL of the developed sample was applied to the receiver compartment plate (Multiscreen™-IP, MAIPN4510, pore size 0.45 µm; Millipore, Merck Ltd., Budapest, Hungary). The sandwich system was incubated at 37 °C for 4 h. After separation of the PAMPA sandwich plates, the concentrations of CBZ in the acceptor solutions were determined. For each assay, three replicates per formulation were measured. The effective permeability and membrane retention of the drugs were calculated using the following equation [46]:

(5) $$P_{e}\;=\;-\frac{2.303\;\times V_{A}}{A\;\left(t\;-\;\tau_{SS}\right)}\times \;\log\;\left[1\;-\;\frac{C_{A}\left(t\right)}{S}\right]$$

where *Pe* is the effective permeability coefficient (cm/s), *A* is the filter area (0.3 cm^2^), *V_A_* is the volume of the acceptor phase (0.3 cm^3^), *t* is the incubation time (s), *τ_SS_* is the time to reach steady state (s), *CA* (*t*) is the concentration of the compound in the acceptor phase at time *t* (mol/cm^3^), and *S* is the solubility of CBZ.

The flux of the samples was calculated using the following equation:

(6) $${\mathrm{Flux}\;=\;P}_{e}\;\times \;S$$

In this study, the PAMPA-BBB assay was used as a standardised, high-throughput screen of passive permeability across a brain-like lipid barrier rather than as a full biological model of the BBB or nose-to-brain transport. Thus, the resulting P_e_ values serve to comparatively rank CBZ permeability between formulations and complement the nasal diffusion data obtained with the cellulose/IPM membrane [47].

### 2.10. Evaluation of Formulated Product Stability

Key factors such as particle size and PDI were evaluated as part of the stability study of the developed formulations under various conditions. The formulations were tested in SNES for one hour to mimic the nasal environment and in PBS (pH 7.4) for one hour to simulate physiological conditions. To assess long-term stability, the developed formulations were stored at 8 °C, with stability monitored over one month.

### 2.11. Statistical Analysis

All experimental data were expressed as the mean ± standard deviation (SD) of at least three independent replicates. Statistical comparisons between the CBZ@BSA NPs and CBZ@SβCD-BSA NPs formulations for physicochemical characteristics (particle size, PDI, DL%, and EE%) were performed using Welch’s *t*-test, which accounts for unequal variances between groups. A *p*-value of less than 0.05 (*p* < 0.05) was considered to indicate a statistically significant difference. The similarity factor (f2) was employed to compare the dissolution profiles of different formulations. An f2 value between 50 and 100 indicates similarity between two dissolution profiles. All statistical analyses, including *t*-tests and f2 calculations, were performed using Python (version 3.11) with the SciPy library (version 1.11.4).

## 3. Results and Discussion

### 3.1. Evaluation of Experimental Design Outcomes

**Figure 2:**
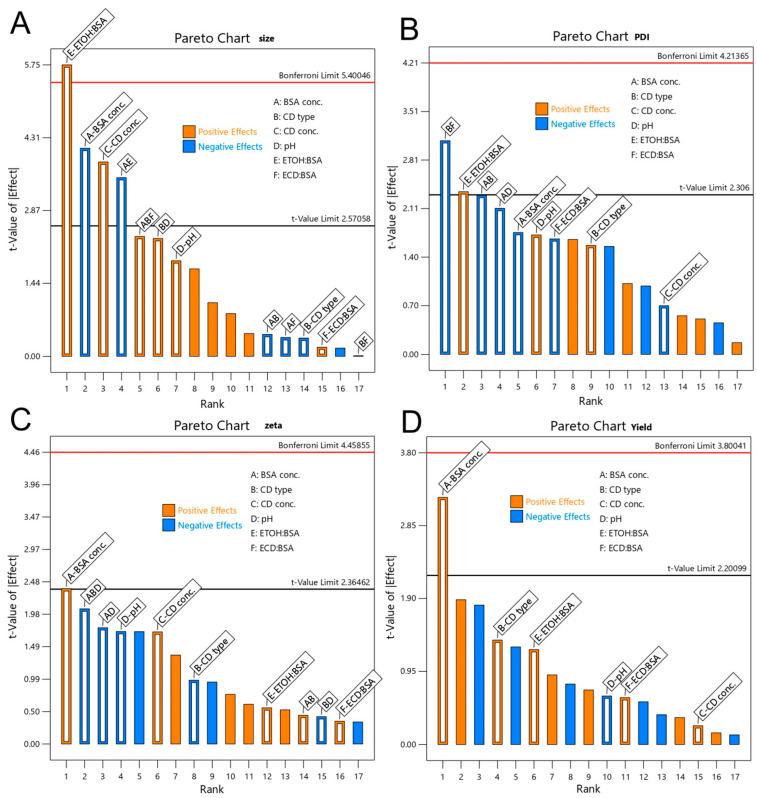
Experimental design results: effect of parameters on particle size (**A**), effect of parameters on PDI (**B**), effect of parameters on zeta potential (**C**) and effect of parameters on nanoparticle yield (**D**). Factors: A = BSA concentration; B = CD type (HβCD vs. SβCD); C = CD concentration; D = pH; E = EtOH:BSA ratio; F = EDC:BSA ratio (see Table 2 for levels).

Examination of the experimental design results shown in Figure 2 revealed a positive correlation between the volume of EtOH and the concentration of the βCD derivative, as well as particle size, indicating that higher values of either parameter produced larger nanoparticles. Conversely, increasing the BSA concentration reduced nanoparticle size, as depicted in Figure 2. This observation aligns with findings from Rahimnejad et al., who reported similar trends within a low BSA concentration range (5–30 mg/mL) [48], and was corroborated by Radwan et al., who confirmed these changes as the BSA concentration rose from 2.5% to 10% [49]. The phenomenon can be explained by the increased availability of BSA molecules, which promote nucleation and result in the formation of more compact nanoparticles that ultimately yield smaller particle sizes. Furthermore, our findings highlight the role of EtOH as a desolvating agent; at low EtOH concentrations, BSA molecules remained largely in the aqueous phase, inhibiting significant nanoparticle formation. As EtOH levels increased, partial desolvation promoted the self-assembly of BSA into loosely organised nanoparticles, which then transformed into more compact forms as EtOH concentrations further increased (Figure S2). This behaviour aligns with previous research on protein nanoparticle formation, where dehydration induced by EtOH promoted molecular aggregation and stabilisation [50].

The βCD derivative type and EDC crosslinker concentration had a negative effect on the PDI, indicating that their presence improved the uniformity of the nanoparticle size distribution (Figure 2). Zeta potential values for all runs remained within the generally accepted stability window (−30 to +30 mV), indicating overall colloidal stability; however, no single factor exceeded the statistical threshold to be considered a dominant determinant of the surface charge (Figure 2). Moreover, yield analysis demonstrated that BSA concentration was crucial; at higher BSA concentrations, nanoparticle recovery was significantly enhanced (Figure 2).

Based on these findings, the βCD derivative type SβCD was selected for further investigation because it showed a better response than HβCD. In addition to the βCD derivative type, SβCD concentration, the EtOH volume, and the BSA concentration were identified as the most critical factors and were subjected to further optimisation in the presence of CBZ. Following the DOE screening study, it was determined that pH had no significant influence on the observed responses. Therefore, for the optimisation study, the pH was maintained at a fixed value (pH 8). This was also supported by the research of Jenita et al., which indicated that BSA tends to generate smaller aggregates at this specific pH level. This phenomenon is attributed to a stronger negative surface charge that mitigates molecular aggregation via electrostatic repulsion [51]. Concerning the crosslinker, while EDC is most effective at acidic pH levels (4.5–6), it can still facilitate crosslinking at elevated pH levels when applied in larger quantities [52]. Accordingly, the EDC concentration was set at 3% (*w*/*w* EDC:BSA) to guarantee sufficient crosslinking efficiency within the chosen pH conditions. As noted earlier, the primary variables identified were factors A, B, C, and E. These factors were chosen for additional optimisation alongside CBZ, as detailed in Table 3.

### 3.2. Optimisation of Formulation

The impact of EtOH volume on the formation of SβCD-BSA nanoparticles was investigated in terms of particle size, EE%, and overall stability. EtOH was added dropwise to BSA:SβCD solutions at ratios of 1:1, 1:2, 1:3, and 1:4 (*v*/*v*) and at molar ratios of 1:1, 1:2, 1:3, and 1:4 (mol/mol). The experimental outcomes confirm the crucial role of EtOH concentration in nanoparticle formation and drug encapsulation.

**Figure 3:**
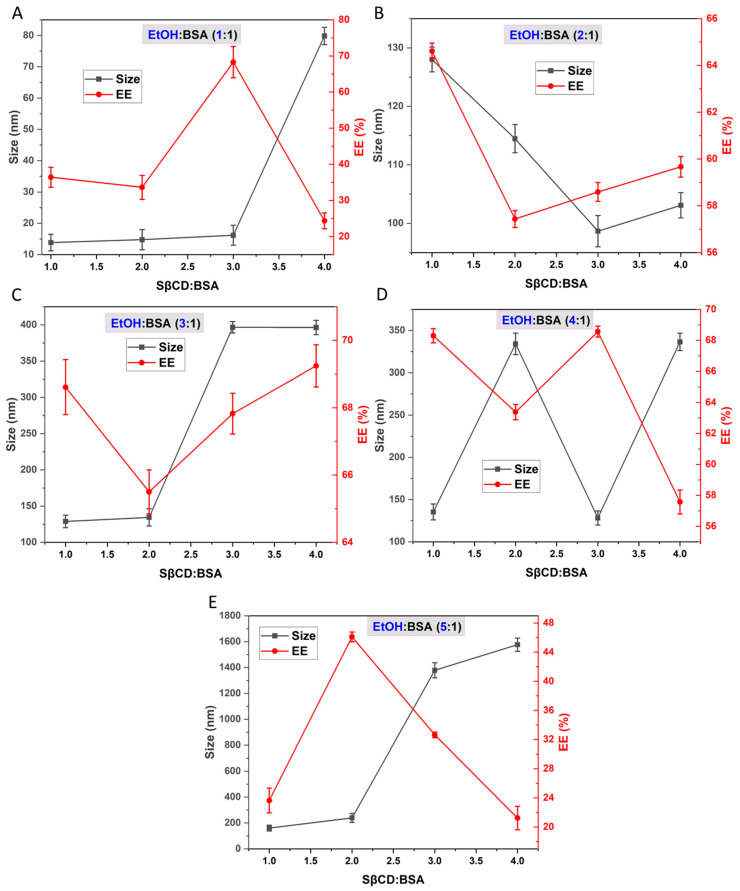
The size and EE% of prepared nanoparticles using different EtOH: BSA ratios (EtOH: BSA 1:1 (**A**); EtOH: BSA 2:1 (**B**); EtOH: BSA 3:1 (**C**); EtOH: BSA 4:1 (**D**); EtOH: BSA 5:1 (**E**)) and varying SβCD: BSA molecular ratios.

At a 1-fold EtOH volume, nanoparticle formation was ineffective; BSA molecules remained in solution rather than forming structured nanoparticles, as mentioned above. This suggests that inadequate EtOH levels limit proper desolvation and self-assembly of the protein into nanoparticles, leading to poor CBZ as well (Figure 3). As the EtOH ratio increased to 2:1 and 3:1 (*v*/*v*), nanoparticle formation was observed, with particle sizes within an acceptable range. EE% improved notably at 2:1 and 3:1 (*v*/*v*) EtOH ratios, likely due to enhanced cavity formation for CBZ entrapment [53]. Nevertheless, at a 3:1 (*v*/*v*) ratio, a gel-like albumin residue accumulated at the tube edges after centrifugation, potentially affecting the reported EE% values (Figure S3).

**Figure 4:**
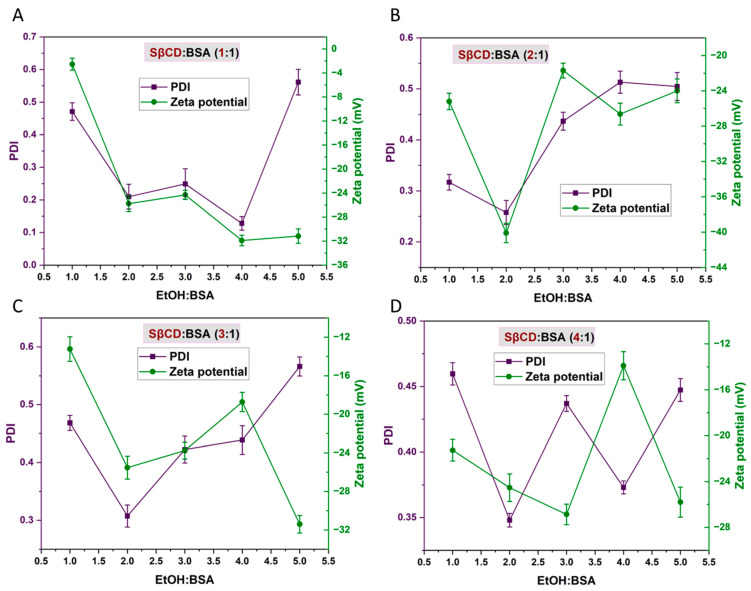
The PDI and zeta potential of prepared nanoparticles using different molecular ratios of SβCD-BSA and varying ratios of EtOH-BSA (EtOH:BSA 1:1 (**A**); EtOH:BSA 2:1 (**B**); EtOH:BSA 3:1 (**C**); and EtOH:BSA 4:1 (**D**)).

A further increase in EtOH volume to 4:1 (*v*/*v*) resulted in larger nanoparticles and a higher PDI, indicating reduced homogeneity (Figure 4). Despite maintaining EE% between 57% and 68%, the presence of protein aggregation and residual gelatinous material suggested partial instability at this concentration. At the highest EtOH volume (5:1 *v*/*v*), nanoparticle formation was significantly hindered, with a maximum EE% of 45% and particle size exceeding the nano range when a higher amount of SβCD was used (Figure 3). Formulations with higher SβCD: BSA ratios (3:1 and 4:1 mol/mol) exhibited substantial precipitation, suggesting that excessive EtOH disrupts nanoparticle integrity, likely due to BSA denaturation [54]. In contrast, formulations with lower SβCD: BSA ratios (1:1 and 2:1 mol/mol) showed acceptable size distribution, but EE% decreased. These findings emphasise the critical role of EtOH in modulating nanoparticle formation and drug encapsulation. Moderate EtOH volumes (2:1 to 3:1 *v*/*v*) appear to promote optimal nanoparticle formation; therefore, SβCD: BSA ratios (1:1 mol/mol) and EtOH volumes (2:1 *v*/*v*) were chosen as the optimal ratios for the subsequent formulation studies.

Regarding PDI, it was observed that a 1-fold EtOH: BSA ratio (*v*/*v*) produced a high PDI across all SβCD: BSA molar ratios (1:1, 2:1, 3:1 and 4:1), as shown in Figure 4. It should be noted that as the SβCD: BSA molar ratio increased, the PDI also increased. The 2-fold EtOH: BSA ratio (*v*/*v*) produced the lowest PDI value (less than 0.33), indicating a uniform particle size distribution. However, as the EtOH amount increased to 3- and 4-fold, the PDI increased to 0.3–0.5, which suggests that excessive EtOH may have disrupted the nanoparticulate system, possibly by altering protein conformation or interfering with stable nanoparticle formation. At the 5-fold level, the system exhibited greater size variability and instability, likely due to aggregation or breakdown of the nanoparticle structure. In connection with zeta potential, values ranged from –2 to –32 mV, which are considered adequate for physical stability and acceptable for brain delivery, as previously discussed.

### 3.3. Determination of Drug Loading (DL%) and Encapsulation Efficiency (EE%)

DL% and EE% play a key role in achieving the therapeutic window; higher values allow a smaller dose to achieve effective plasma concentrations [55]. The SβCD-BSA nanoparticles exhibited a statistically significant higher DL% and EE% (*p* = 0.0106 and *p* = 0.0101, respectively) compared to the non-functionalized nanoparticles (Table 4; Table S3). This suggests that SβCD significantly enhances CBZ entrapment, possibly by reducing drug crystallisation and improving interaction with the BSA matrix [56].

### 3.4. The Effect of SβCD and EDC on the Colloidal Parameters and Encapsulation Efficiency (EE%)

**Figure 5:**
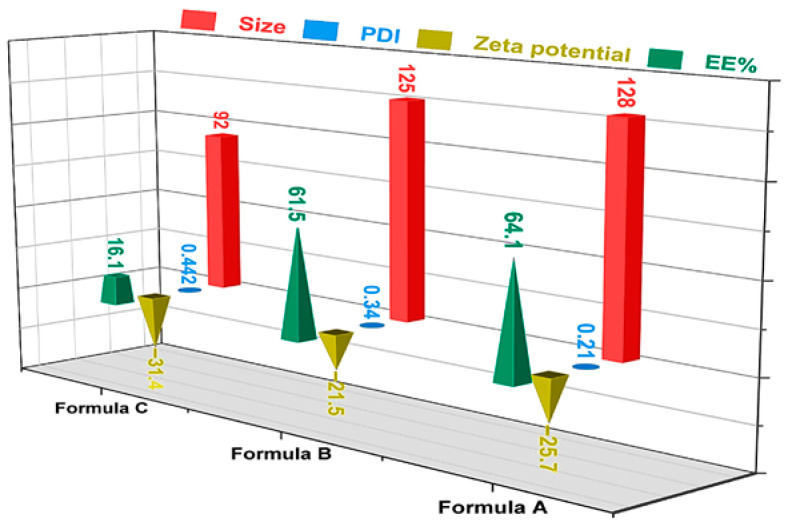
The recorded responses with respect to the presence or absence of SβCD or EDC: Formula A: CBZ@SβCD-BSA (the optimal formula with SβCD and EDC crosslinker); Formula B: CBZ@BSA (excluding only SβCD); and Formula C: CBZ@SβCD-BSA (excluding only EDC).

To confirm the essential role of SβCD and EDC in achieving optimal formulation characteristics, comparative characterisations were performed using nanoparticulate systems containing SβCD, EDC, or both, using the optimal factors and their levels (Figure 5). While the individual contribution of SβCD to the formulation showed no statistically significant changes in particle size or PDI, it resulted in a significant increase in EE% (Table S3). This increase can be attributed to the additional CBZ molecules included in the complexes with Sβcd. The rise in size may be due to SβCD’s ability to interact with BSA, encouraging or stabilising self-assembly by forming inclusion complexes or via electrostatic and hydrophobic interactions [57].

The lower PDI can be explained by this functional group’s ability to prevent nanoparticle aggregation. However, gel formation, an indication of stronger protein–protein interactions, occurred at lower EtOH concentrations in the only BSA system, whereas it was delayed in the presence of SβCD and emerged only at a higher EtOH ratio.

At a fixed EtOH-to-BSA ratio (2:1 *v*/*v*), the particle size increased slightly from 92 to 128 nm when EDC was used as a crosslinker (Figure 5). Most importantly, EDC stabilised the particle size across different EtOH concentrations, as indicated by the lower PDI values in the presence of EDC. Furthermore, the EDC-containing formulation showed significantly higher EE% than the other formulations. One potential reason for this enhancement is that EDC enables the intra- and inter-molecular crosslinking of BSA, which leads to a more prolonged retention of the drug and minimises CBZ leakage during nanoparticle development (Figure 5). Crosslinked BSA nanoparticles exhibit a less negative zeta potential than their respective non-crosslinked variants, indicating a shift in surface charge. This shift is ascribed to the neutralisation of surface carboxyl groups via the formation of amide bonds, leading to a decrease in the net negative charge on the surface of the nanoparticles.

### 3.5. Morphological Characterisation (SEM)

**Figure 6:**
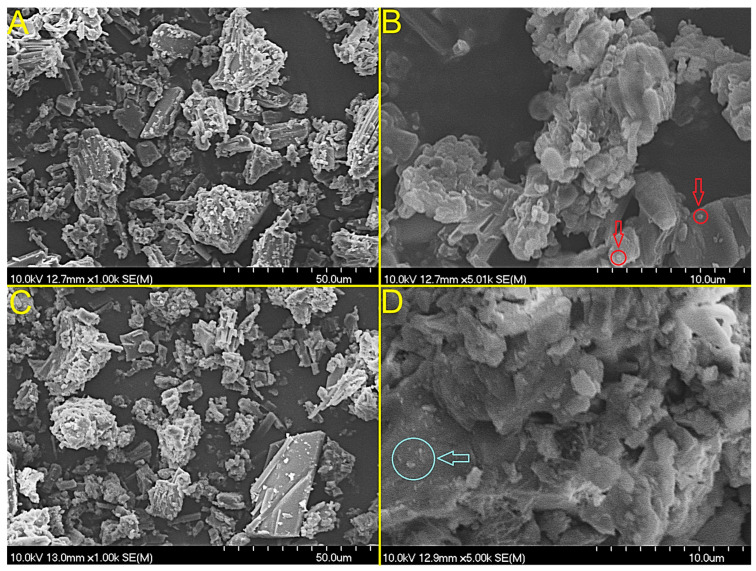
Morphological characterisation of freeze-dried nanoparticles using SEM exhibiting a homogeneous surface with fused nanoparticle aggregates: CBZ@BSA (**A**,**B**) and CBZ@SβCD-BSA (**C**,**D**). Arrows indicate the individual spherical nanoparticles.

The surface morphology of the freeze-dried CBZ@BSA and CBZ@SβCD-BSA nanoparticle powders was examined using SEM (Figure 6). As depicted in Figure 6, the micrographs revealed a porous, flake-like aggregate structure rather than discrete individual nanoparticles. This morphology is characteristic of lyophilized protein-based nanocarriers protected with cryoprotectants (e.g., mannitol), where individual spherical nanoparticles (confirmed by DLS to be ~128 nm) cluster together during the freezing and drying process to form larger micro-scale assemblies [58]. The observed porous structure suggests the successful sublimation of ice crystals during lyophilization, which facilitates rapid reconstitution upon administration. At higher magnifications (up to 10,000×), the surface appeared relatively homogeneous without significant drug crystallisation, indicating uniform drug distribution within the BSA matrix [59]. The structural integrity remained intact for both formulations, with no distinct morphological differences observed between the non-functionalized and SβCD-functionalized samples, suggesting that the functionalization process did not compromise the physical stability of the nanocarrier matrix.

### 3.6. FTIR Structural Investigation

**Figure 7:**
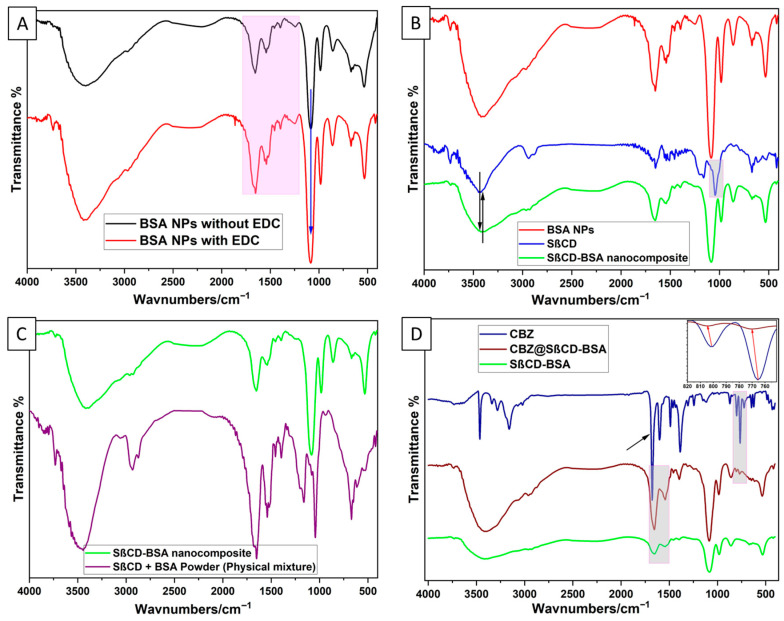
FTIR spectra of BSA nanoparticles with and without EDC (**A**); SβCD-BSA NPs (**B**); physical mixture of SβCD and BSA (**C**); and free CBZ compared with incorporated CBZ (**D**).

This study examined the FTIR spectra of EDC-crosslinked and non-crosslinked BSA, SβCD, and CBZ. Characteristic FTIR spectral peaks for BSA appeared at 857, 982, 1083, 1244, 1543, 1655, 2963, and 3398 cm^−1^ (Figure 7A). The spectra remained unchanged with the addition of EDC, indicating that EDC, functioning as a zero-length crosslinker, does not integrate into the final product but promotes the direct linking of carboxylate groups (–COOH) to primary amines (–NH_2_) (Figure S4) [60]. The peak at 1655 cm^−1^ corresponds to the amide I band, associated with C=O stretching vibrations and the α-helix structure of proteins. The peak at 1543 cm^−1^ signifies the amide II band and involves N–H bending along with C–N stretching [61]. The peak at 1244 cm^−1^ is linked to the amide III band, and the 2963 cm^−1^ peaks are related to aliphatic C–H stretching [62]. A broad peak at 3398 cm^−1^ results from O–H stretching. The FTIR spectrum of SβCD features a significant band centred at 3426 cm^−1^ due to strong O–H stretching vibrations from primary or secondary hydroxyl groups. Additionally, a moderate peak at 2938 cm^−1^ reflects C–H stretching, and a pronounced band at 1043 cm^−1^ arises from the O–H bending vibration.

The functionalization of BSA nanoparticles with SβCD results in a pronounced shift in the O–H stretching band from 3426 cm^−1^ to 3402 cm^−1^, accompanied by the disappearance of the 1043 cm^−1^ peak. These changes indicate alterations in the hydrogen-bonding environment, suggesting interactions with hydroxyl groups that may facilitate new bond formation or structural rearrangements within the samples (Figure 7B). The mechanism of functionalization was further confirmed by examining the FTIR spectra of the SβCD and BSA nanoparticle physical mixture alongside those of the hybrid formulation. Notably, the physical blend exhibited distinct peaks for each component without any evident changes, suggesting no interactions or bond formation between the components in their unaltered form (Figure 7C).

**Figure 8:**
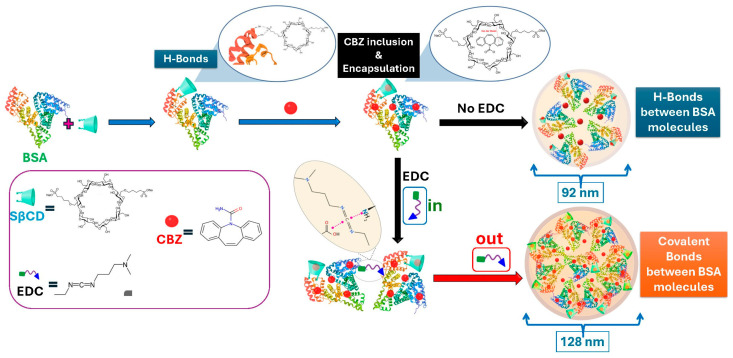
The mechanism of nanocomposite formation.

In the FTIR spectrum of CBZ, a narrow band at 3465 cm^−1^ indicates N–H stretching from the primary amine group. Aromatic C–H stretching vibrations appear at 3160 cm^−1^, and the peak at 1387 cm^−1^ corresponds to N–H deformation. The C=C ring-stretching vibration, characteristic of the compound, occurs at 1489 cm^−1^, and a medium-intensity band at 1677 cm^−1^ is due to the C=O bond in the amide group. Moreover, the principal peaks for aromatic ring hydrogens are located at 800 and 765 cm^−1^ [63,64]. The comparison of the FTIR spectra of pure CBZ and the CBZ@SβCD-BSA composite reveals significant changes indicative of interactions and structural modifications (Figure 6D). The peak positions for the amide I (α-helix) and amide II bonds of BSA shifted from 1655 to 1652 cm^−1^ and from 1548 to 1544 cm^−1^, respectively, suggesting that the secondary structure of BSA was perturbed upon CBZ binding [56,65]. The principal peaks in the hydrogens of the aromatic ring at 800 and 765 cm^−1^ in pure CBZ redshifted to 805 and 771 cm^−1^, respectively. The absence of HC=CH and –C=O groups in the CBZ spectrum within the composite indicates hydrophobic inclusion/encapsulation of CBZ within the SβCD-BSA composite [64]. These spectral changes collectively demonstrate the successful incorporation of CBZ into the composite structure, altering its environment and interactions [56,65]. A schematic illustration of the mechanism of nanocomposite formation, based on FTIR and previous results, is shown in Figure 8.

### 3.7. In Vitro Assessment Results

#### 3.7.1. Investigation of the Mucoadhesive Properties

**Figure 9:**
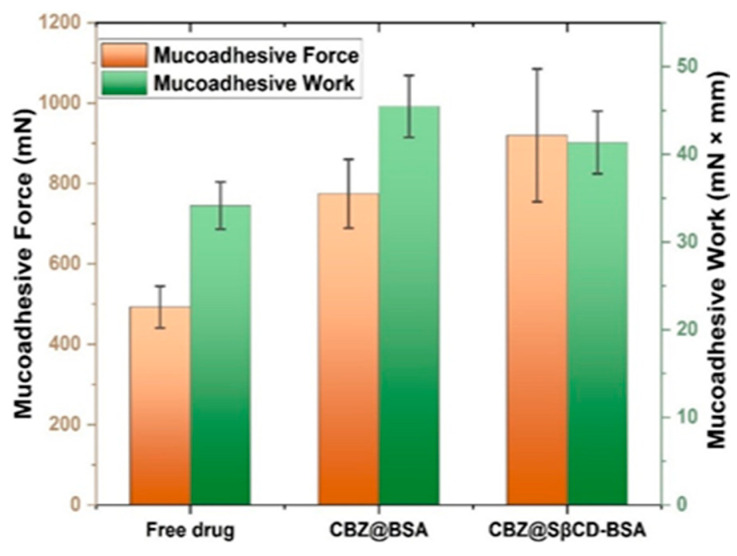
Mucoadhesive result for CBZ suspension, and the developed formulations.

The initial barrier to the intranasal route is the negatively charged, porous mucus layer, which mainly consists of mucin polysaccharides. This barrier is reinforced by mucociliary clearance, with an estimated half-life of 15–30 min. To minimise the effect of mucociliary clearance (MCC) and maximise residence time, SβCD was also included in the formulation, as it increases viscosity and permeability [66]. In this study and the following studies, mucoadhesion was investigated in the pristine, unformulated form, CBZ@BSA, and in the CBZ@SβCD-BSA formulations (Figure 9). Interestingly, SβCD exhibited a dual effect: it increased adhesive force by approximately 2-fold compared with the free drug, while simultaneously decreasing adhesive work compared with CBZ@BSA. Values were 34.2 ± 2.45, 41.3 ± 3.25, and 45.5 ± 3.22 mN × mm for the free drug, CBZ@BSA, and CBZ@SβCD-BSA, respectively. As a reference, 0.5% *w*/*w* low-molecular-weight (1400 kDa) sodium hyaluronate (NaHA) was selected as a frequently used mucoadhesive excipient in nasal formulations. Texture analyzer measurements of 0.5% *w*/*w* NaHA showed 62.88 ± 20.94 mN × mm adhesive work, indicating no significant difference compared with CBZ@SβCD-BSA. This confirms that CBZ@SβCD-BSA and 0.5% *w*/*w* NaHA have similar mucoadhesive properties. The increase in adhesive force can be attributed to electrostatic and hydrophobic interactions between the formulation and the mucosal barrier.

Formulations incorporating SβCD exhibited a significantly higher adhesive force, contrary to the expected charge repulsion arising from the negatively charged nature of SβCD relative to the mucus layer. This observation implies that other interactions, including hydrogen bonding, dipole–dipole interactions, hydrophobic interactions, and van der Waals forces, play a crucial role in maintaining the formulation’s presence on the mucosal surface [67,68]. Notably, these formulations demonstrated an extended retention time within the nasal cavity, which likely facilitated improved absorption across the membrane. In contrast, the negative charge associated with SβCD reduced adhesive work compared with formulations devoid of it. This decrease in adhesive work can be attributed to a faster detachment mechanism, in which repulsive forces effectively neutralise the overall adhesion strength, resulting in easier displacement upon the application of mechanical stress. This finding emphasises the complex role of SβCD in modulating mucoadhesion and suggests that achieving a careful equilibrium between adhesive strength and work performed is essential for optimising intranasal drug delivery efficacy.

#### 3.7.2. In Vitro Drug Release in SNES and PBS

**Figure 10:**
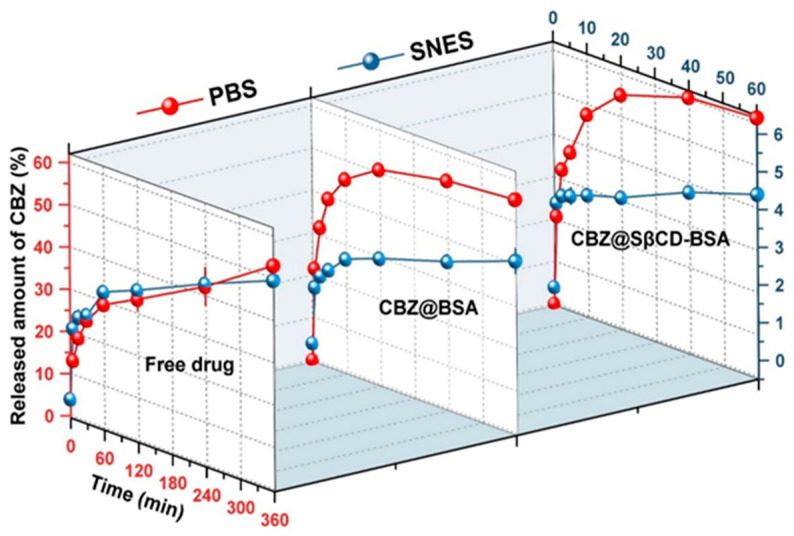
Release profile of CBZ from CBZ@BSA and CBZ@SβCD-BSA compared to the free drug in SNES and in PBS.

The free drug formulation exhibits the fastest release profile among the developed formulations, with approximately 6% ± 0.17, 3.7 ± 0.49 and 4.3 ± 0.019 for free drug, CBZ@BSA and CBZ@SβCD-BSA, respectively (Figure 10). This can be explained by the presence of CBZ within the BSA matrix, which acts as a barrier that delays and reduces CBZ release. The nanoparticles made from BSA grafted with SβCD display a release profile that is intermediate compared with the other two formulations discussed earlier. Here, SβCD seems to improve the solubility of CBZ while facilitating its release from the BSA matrix. These results indicate that the developed formulations encapsulate CBZ, preventing its release in the nasal cavity while preserving it for release at later stages.

By contrast, the release profiles in SNES reveal that free CBZ exhibits slow and incomplete dissolution (≈53% ± 0.61) due to its poor aqueous solubility. After 360 min, only ≈53 ± 0.61% is released (Figure 10). Conversely, CBZ@BSA nanoparticles exhibit a biphasic release, with an initial burst (≈50% in the first hour) followed by sustained release, likely due to surface-bound drug desorption and core diffusion. The CBZ@SβCD-BSA formulation demonstrates the highest release efficiency, reaching approximately 65%, suggesting that SβCD enhances CBZ solubility and diffusion by reducing drug–protein interactions and CBZ inclusion in its cavities.

These results are consistent with earlier findings indicating a biphasic release profile for CBZ-loaded nanoparticles. Jain et al. reported that an SβCD inclusion complex markedly improved CBZ dissolution compared with CBZ alone, with a rapid initial burst release due to adsorbed drug on the particle surface, followed by sustained drug release, as drug diffused from the nanoparticle core [64]. Likewise, Erukula et al. reported a biphasic release of CBZ from human serum albumin nanoparticles, with an initial burst phase lasting up to four hours [56]. The solubility-enhancing effects of SβCD were further demonstrated by Smith et al., who reported that solubility increased from 0.1 mg/mL to 5.4 mg/mL due to the formation of an inclusion complex, thereby improving both dissolution and bioavailability [69]. Additionally, Volkova et al. highlighted that increasing SβCD concentration enhances CBZ solubility and permeation, likely due to its high affinity for CBZ and improved membrane permeability, a key factor in drug absorption [70].

It is worth noting that the lower CBZ release from the two developed formulations in the SNES medium compared with the free drug may be due to the presence of calcium ions (Ca^2+^), which can stabilise albumin nanoparticles by forming crosslinks between protein molecules, thereby slowing drug release [71].

On the other hand, in PBS medium, where calcium is absent, the nanoparticles may be less stable, allowing more drug to be released. Also, the presence of SBE-β-cyclodextrin in the formulation increases the water solubility of CBZ, resulting in higher release than that of the free drug in PBS [69].

To quantitatively compare the release profiles, the similarity factor (f2) was calculated. The f2 value between CBZ@BSA and CBZ@SβCD-BSA profiles in PBS was 69.74, indicating that the release kinetics are similar. Furthermore, the f2 values comparing the nanoparticle formulations to the free drug were below 50 (44.17 for CBZ@BSA and 40.84 for CBZ@SβCD-BSA), confirming that both nanoparticle systems provide a dissimilar, more controlled release pattern (Table S4).

#### 3.7.3. In Vitro Horizontal Diffusion Study

**Figure 11:**
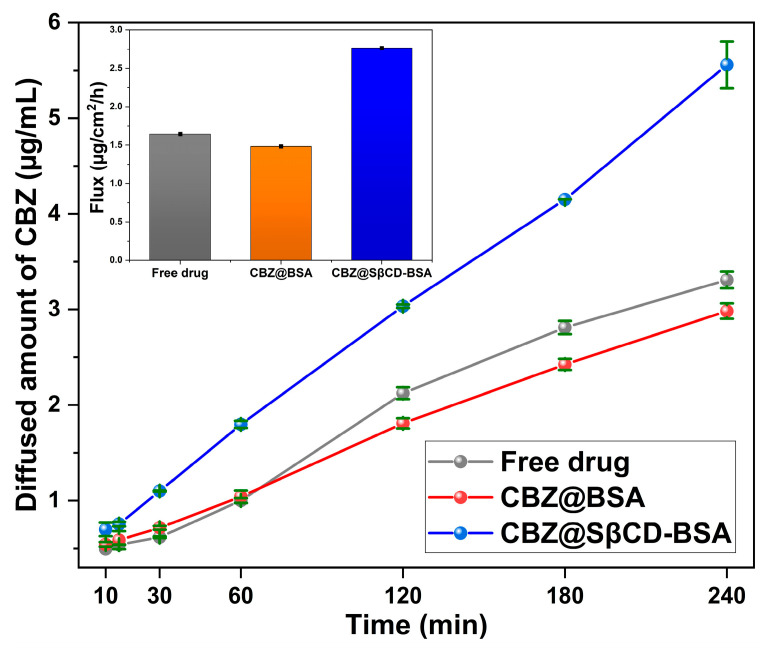
Horizontal diffusion of CBZ from CBZ@BSA and CBZ@SβCD-BSA compared to the free drug.

All formulations exhibited time-dependent diffusion patterns, as shown in Figure 11. Furthermore, we observed considerable variability in both the rate and extent of diffusion across groups. CBZ@SβCD-BSA produced the highest flux (2.7 µg/cm^2^/h), nearly double that of CBZ@BSA, indicating ease of permeation due to SβCD (Figure 11 inset). This improvement may be due to SβCD’s higher solubility, smaller particle size, or permeability-enhancing properties. In comparison, the CBZ suspension showed moderate diffusion performance, and the lowest rate and flux were observed with CBZ@BSA nanoparticles. This reduced performance can be attributed to tighter matrix entrapment.

#### 3.7.4. BBB-PAMPA

**Figure 12:**
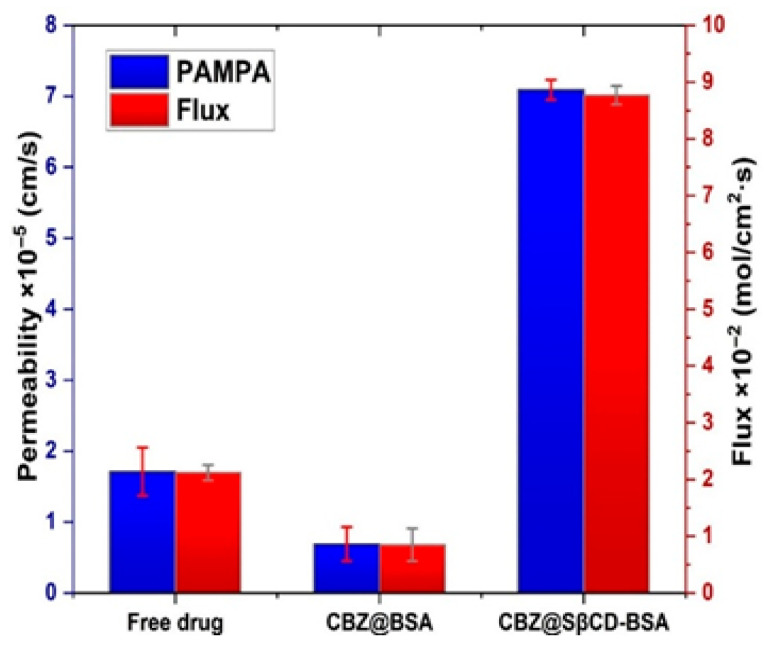
PAMPA permeability and flux of CBZ from CBZ@BSA and CBZ@SβCD-BSA compared to free drug.

The permeability and flux results from the PAMPA in Figure 12 reveal the significant superiority of CBZ@SβCD-BSA over the free drug and CBZ@BSA, with 2-fold and 10-fold increases, respectively. The CBZ@SβCD-BSA formulation exhibited permeability and flux values of 7.9 × 10^−5^ cm/s and 8 × 10^−2^ mol/cm^2^ × s, respectively. This affirms that SβCD enhances CBZ transport across the artificial BBB membrane, again via improving drug solubility and reducing intermolecular interactions. In contrast, the free CBZ suspension displayed moderate permeability and flux, though with noticeable variability, suggesting inconsistent drug solubilization and diffusion rates. The CBZ@BSA formulation displayed the lowest permeability and flux, indicating that excessive BSA might hinder CBZ diffusion, possibly due to strong drug–protein interactions that limit the availability of free drug molecules for passive transport [72].

### 3.8. Stability Studies

The data confirm that both CBZ@BSA and CBZ@SβCD-BSA formulations showed a slight increase in size and PDI immediately after lyophilization (0.25 ± 0.06 and 0.31 ± 0.09 for CBZ@SβCD-BSA and CBZ@BSA, respectively). However, these changes remained within acceptable limits, indicating that no irreversible process leading to loss of colloidal stability occurred. The particle size increased slightly during the hour of dispersion in SNES, supporting sufficiently stable behaviour under nasal-like conditions. In PBS (pH 7.4), a more substantial increase in particle size over the same time period was observed, which may also be linked to the higher drug release in this medium (Table 5).

After one month of storage, both formulations showed an insignificant increase in particle size, with values of 179.15 ± 2.93 and 191.53 ± 2.19 for CBZ@SβCD-BSA and CBZ@BSA, respectively. Nevertheless, the particles in CBZ@BSA were larger, suggesting the stabilising role of SβCD against aggregation. Under these conditions, the CBZ@SβCD-BSA formulation generally exhibited lower PDI values, at 0.32 ± 0.11 and 0.35 ± 0.09 for CBZ@SβCD-BSA and CBZ@BSA, respectively, indicating that SβCD results in improved colloidal stability and a more monodisperse particle size distribution.

### 3.9. Study Limitations

Although this study demonstrates promising physicochemical properties and in vitro performance of CBZ@SβCD-BSA nanoparticles for nasal delivery, several limitations should be appreciated. First, the current models employed simplified artificial membranes (cellulose/isopropyl myristate and PAMPA) rather than biomimetic olfactory cell lines (e.g., RPMI 2650 or olfactory ensheathing cells) or ex vivo porcine nasal mucosa, limiting direct assessment of olfactory region penetration and nose-to-brain translocation efficiency. In addition, the present work considers intranasal administration at the level of the whole nasal cavity rather than a specifically engineered deposition into the olfactory region, so the fraction of the administered dose that actually reaches the olfactory mucosa cannot be quantified from these data. Second, nasal epithelial cytotoxicity has not been evaluated. Finally, in vivo pharmacokinetics, brain targeting efficiency, and toxicology data are needed to validate therapeutic translation and safety. These limitations highlight the need for advanced ex vivo and in vivo validation studies in future research.

## 4. Conclusions

This study successfully developed and characterised CBZ@SβCD-BSA nanoparticles optimised via fractional factorial design for nasal delivery. The formulation exhibited desirable physicochemical properties (hydrodynamic size: 180 ± 12 nm; PDI: 0.18 ± 0.04; ζ-potential: −28 ± 3 mV), high drug loading (DL%: 13.1 ± 1.5%; EE%: 82.5 ± 4.2%), and superior in vitro performance compared to free CBZ and CBZ@BSA: enhanced nasal diffusion (flux 2.3-fold higher), favourable PAMPA-BBB permeability (P_e = 4.2 × 10^−6^ cm/s), and 4-week stability.

## Figures and Tables

**Table 1:** Summary of previous nanotechnology-based formulations of carbamazepine for enhanced delivery.

Study/Author	Nanocarrier Type	Route/Target	Key Size/PDI/ζ-Potential	Entrapment/Release Behaviour	Main Outcome	Ref.
Qushawy et al.	Solid lipid nanoparticles (SLNs)	Likely systemic/brain	Not specified; SLNs	EE ≈ 40–72%; controlled release	Enhanced anticonvulsant activity and improved brain protection vs. conventional CBZ.	[26]
Ana et al.	Polymer–lipid hybrid NPs, chitosan-coated	Intestinal permeability	Size ≈ 150 nm; very low PDI (<0.150)	Not specified; focus on permeability	Markedly increased intestinal permeability.	[8]
Liu et al.	Carboxymethyl chitosan nanoparticles	Intranasal; BBB/brain	Not specified	Not specified	Intranasal NPs penetrated BBB and enhanced brain targeting.	[27]
Montoto et al.	SLNs and nanostructured lipid carriers (NLCs)	Systemic/brain	Not specified	Controlled release from SLNs/NLCs	Prolonged in vivo seizure protection.	[28]
Kandilli et al.	PLGA NPs co-loaded with CBZ and levetiracetam	Likely systemic/brain	Size ≈ 181 nm; ζ ≈ −27 mV	EE not fully detailed; biphasic CBZ release (≈90% in 2 days); rapid LEV release (≈80% in 30 min)	Combined therapy with controlled CBZ and fast LEV release.	[29]

**Table 2:** Independent factors and their levels in the screening design.

	Independent Variable	Coded Level	
		−1	+1
A	BSA con. (mg)	10	30
B	CD type	HβCD	SβCD
C	CD conc. (mg)	1	10
D	pH	7	9
E	EtOH: BSA (*v*/*v*)	1	6
F	EDC: BSA (%)	1	5

**Table 3:** Variables used in the optimisation of the formulation in the presence of CBZ.

Factors	Range/Value
βCD type	SβCD
SβCD: BSA molecular ratio	1–4
ETOH: BSA *v*/*v*	1–5
pH	8
EDC: BSA %	3

**Table 4:** The EE% and DL of the developed formulas.

Formulation	DL%	EE%
CBZ@BSA	31.91 ± 1.50	38.10 ± 1.58
CBZ@SβCD-BSA	34.28 ± 1.60	41.01 ± 2.55
(*p*-value *)	0.0106	0.0101

* Welch’s *t*-test comparing CBZ@SβCD-BSA to CBZ@BSA.

**Table 5:** Results of the stability study.

Formula	Medium	Time (h)	Size (nm)	PDI
CBZ@BSA	PBS	0 h	116 ± 2.13	0.33 ± 0.04
		1 h	125 ± 1.25	0.31 ± 0.07
	SNES	0	127 ± 1.12	0.28 ± 0.09
		1 h	121 ± 2.38	0.26 ± 0.12
	Lyophilized powder	1 month	191 ± 2.19	0.35 ± 0.09
CBZ@SβCD-BSA	PBS	0 h	130 ± 1.21	0.29 ± 0.05
		1 h	145 ± 1.98	0.25 ± 0.15
	SNES	0 h	131 ± 1.75	0.31 ± 0.10
		1 h	126 ± 2.37	0.23 ± 0.12
	Lyophilized powder	1 month	179 ± 2.93	0.32 ± 0.11

## Data Availability

The original contributions presented in this study are included in the article/Supplementary Material. Further inquiries can be directed to the corresponding author.

## Supplementary Materials

The following supporting information can be downloaded at: https://www.mdpi.com/article/10.3390/pharmaceutics18030331/s1, Figure S1: Carbamazepine Market Report about the global Carbamazepine market size in USD in 2025.; Figure S2: Illustration of the formation mechanism of BSA NPs by the desolvation process.; Figure S3: The qualitative gel residue on the edge of the tube after centrifuging.; Figure S4: The mechanism of EDC as cross linker; Table S1: The computer generated 18 experimental runs (factor 2 (B) CD type −1 means HβCD and +1 SβCD); Table S2. DOE Response Surface Models; Table S3. Statistical comparison of physicochemical properties between CBZ@BSA NPs and CBZ@SβCD-BSA NPs; Table S4. Similarity factor (f2) for the in vitro release profiles of CBZ formulations.

## Abbreviations

The following abbreviations are used in this manuscript:

ACN	acetonitrile
AEDs	antiepileptic drugs
APIs	active pharmaceutical ingredients
BBB	blood–brain barrier
BSA	Bovine serum albumin
BSA NPs	bovine serum albumin nanoparticles
CAGR	Compound Annual Growth Rate
CBZ	carbamazepine
CBZ@BSA	carbamazepine-loaded bovine serum albumin nanoparticles
CBZ@HβCD-BSA	carbamazepine-loaded hydroxypropyl-β-cyclodextrin–functionalized BSA nanoparticles
CBZ@SβCD-BSA	carbamazepine-loaded sulfobutyl-β-cyclodextrin–functionalized BSA nanoparticles
CDs	cyclodextrins
CNS	central nervous system
DL%	drug-loading ratio
DOE	Design of experiment
EDC	1-ethyl-3-(3-dimethylaminopropyl) carbodiimide hydrochloride
EE%	encapsulation efficiency
EtOH	Ethanol 96% *v*/*v*
HPLC	High performance liquid chromatography
HβCD	hydroxypropyl-β-cyclodextrin
KBr	potassium bromide
LEV	levetiracetam
MCC	mucociliary clearance
NaHA	sodium hyaluronate
NLCs	nanostructured lipid carriers
PAMPA	Parallel artificial membrane permeability assay
Papp	apparent permeability
PDI	polydispersity index
PLGA	Poly(lactic-co-glycolic acid)
RES	reticuloendothelial system
SLNs	solid lipid nanoparticles
SNES	Simulated nasal electrolyte solution
SβCD	sulfobutyl-β-cyclodextrin
Z-average	average hydrodynamic diameter
ζ-potential	zeta potential

## References

[1] Perucca P., Scheffer I.E., Kiley M. (2018). The management of epilepsy in children and adults. Med. J. Aust..

[2] Neri S., Mastroianni G., Gardella E., Aguglia U., Rubboli G. (2022). Epilepsy in neurodegenerative diseases. Epileptic Disord..

[3] Pong A.W., Xu K.J., Klein P. (2023). Recent advances in pharmacotherapy for epilepsy. Curr. Opin. Neurol..

[4] Beydoun A., DuPont S., Zhou D., Matta M., Nagire V., Lagae L. (2020). Current role of carbamazepine and oxcarbazepine in the management of epilepsy. Seizure.

[5] https://www.prof-research.com/Carbamazepine-Market.

[6] Tsze D.S., Ieni M., Fenster D.B., Babineau J., Kriger J., Levin B., Dayan P.S. (2017). Optimal Volume of Administration of Intranasal Midazolam in Children: A Randomized Clinical Trial. Ann. Emerg. Med..

[7] Poka M.S., Milne M., Wessels A., Aucamp M. (2025). An Investigation into the Effect of Maltitol, Sorbitol, and Xylitol on the Formation of Carbamazepine Solid Dispersions Through Thermal Processing. Pharmaceutics.

[8] Ana R., Mendes M., Sousa J., Pais A., Falcão A., Fortuna A., Vitorino C. (2019). Rethinking carbamazepine oral delivery using polymer-lipid hybrid nanoparticles. Int. J. Pharm..

[9] Illum L. (2003). Nasal drug delivery—Possibilities, problems and solutions. J. Control. Release.

[10] Costa C.P., Moreira J.N., Sousa Lobo J.M., Silva A.C. (2021). Intranasal delivery of nanostructured lipid carriers, solid lipid nanoparticles and nanoemulsions: A current overview of in vivo studies. Acta Pharm. Sin. B.

[11] Agrawal M., Saraf S., Saraf S., Antimisiaris S.G., Chougule M.B., Shoyele S.A., Alexander A. (2018). Nose-to-brain drug delivery: An update on clinical challenges and progress towards approval of anti-Alzheimer drugs. J. Control. Release.

[12] Pires P.C., Rodrigues M., Alves G., Santos A.O. (2022). Strategies to Improve Drug Strength in Nasal Preparations for Brain Delivery of Low Aqueous Solubility Drugs. Pharmaceutics.

[13] Safarov R., Fedotova O., Uvarova A., Gordienko M., Menshutina N. (2024). Review of Intranasal Active Pharmaceutical Ingredient Delivery Systems. Pharmaceuticals.

[14] Shetty A., Keerikkadu M., Bangera P.D., Tippavajhala V.K., Rathnanand M. (2025). An overview of advanced nanocarrier systems for Ibrutinib delivery: Overcoming pharmacokinetic barriers and enabling targeted cancer therapy. Int. J. Pharm. X.

[15] Mohammad H., Darwish M., Katona G., Csóka I. (2025). Functionalized albumin nanoparticles: A multifunctional platform for enhanced brain drug delivery. Mater. Today Bio.

[16] Marcello E., Chiono V. (2023). Biomaterials-Enhanced Intranasal Delivery of Drugs as a Direct Route for Brain Targeting. Int. J. Mol. Sci..

[17] Acosta E. (2009). Bioavailability of nanoparticles in nutrient and nutraceutical delivery. Curr. Opin. Colloid Interface Sci..

[18] Kulkarni S.A., Feng S.-S. (2013). Effects of Particle Size and Surface Modification on Cellular Uptake and Biodistribution of Polymeric Nanoparticles for Drug Delivery. Pharm. Res..

[19] Koo J., Lim C., Oh K.T. (2024). Recent advances in intranasal administration for brain-targeting delivery: A comprehensive review of lipid-based nanoparticles and stimuli-responsive gel formulations. Int. J. Nanomed..

[20] Ribovski L., Hamelmann N.M., Paulusse J.M.J. (2021). Polymeric Nanoparticles Properties and Brain Delivery. Pharmaceutics.

[21] Zhang L., Fan J., Li G., Yin Z., Fu B.M. (2020). Transcellular Model for Neutral and Charged Nanoparticles Across an In Vitro Blood-Brain Barrier. Cardiovasc. Eng. Technol..

[22] Ribeiro M.M., Domingues M.M., Freire J.M., Santos N.C., Castanho M.A. (2012). Translocating the blood-brain barrier using electrostatics. Front. Cell. Neurosci..

[23] Lockman P.R., Koziara J.M., Mumper R.J., Allen D.D. (2004). Nanoparticle Surface Charges Alter Blood–Brain Barrier Integrity and Permeability. J. Drug Target..

[24] Wang G., Siggers K., Zhang S., Jiang H., Xu Z., Zernicke R.F., Matyas J., Uludağ H. (2008). Preparation of BMP-2 containing bovine serum albumin (BSA) nanoparticles stabilized by polymer coating. Pharm. Res..

[25] Roser M., Fischer D., Kissel T. (1998). Surface-modified biodegradable albumin nano- and microspheres. II: Effect of surface charges on in vitro phagocytosis and biodistribution in rats. Eur. J. Pharm. Biopharm..

[26] Qushawy M., Prabahar K., Abd-Alhaseeb M., Swidan S., Nasr A. (2019). Preparation and Evaluation of Carbamazepine Solid Lipid Nanoparticle for Alleviating Seizure Activity in Pentylenetetrazole-Kindled Mice. Molecules.

[27] Liu S., Yang S., Ho P.C. (2018). Intranasal administration of carbamazepine-loaded carboxymethyl chitosan nanoparticles for drug delivery to the brain. Asian J. Pharm. Sci..

[28] Scioli Montoto S., Sbaraglini M.L., Talevi A., Couyoupetrou M., Di Ianni M., Pesce G.O., Alvarez V.A., Bruno-Blanch L.E., Castro G.R., Ruiz M.E. (2018). Carbamazepine-loaded solid lipid nanoparticles and nanostructured lipid carriers: Physicochemical characterization and in vitro/in vivo evaluation. Colloids Surf. B Biointerfaces.

[29] Kandilli B., Ugur Kaplan A.B., Cetin M., Taspinar N., Ertugrul M.S., Aydin I.C., Hacimuftuoglu A. (2020). Carbamazepine and levetiracetam-loaded PLGA nanoparticles prepared by nanoprecipitation method: In vitro and in vivo studies. Drug Dev. Ind. Pharm..

[30] Qu N., Song K., Ji Y., Liu M., Chen L., Lee R.J., Teng L. (2024). Albumin Nanoparticle-Based Drug Delivery Systems. Int. J. Nanomed..

[31] Merkus F.W.H.M., Verhoef J.C., Marttin E., Romeijn S.G., van der Kuy P.H.M., Hermens W.A.J.J., Schipper N.G.M. (1999). Cyclodextrins in nasal drug delivery. Adv. Drug Deliv. Rev..

[32] Pangua C., Espuelas S., Simón J.A., Álvarez S., Martínez-Ohárriz C., Collantes M., Peñuelas I., Calvo A., Irache J.M. (2025). Enhancing bevacizumab efficacy in a colorectal tumor mice model using dextran-coated albumin nanoparticles. Drug Deliv. Transl. Res..

[33] Ramos R., Bernard J., Ganachaud F., Miserez A. (2022). Protein-Based Encapsulation Strategies: Toward Micro-and Nanoscale Carriers with Increased Functionality. Small Sci..

[34] Dhuria S.V., Hanson L.R., Frey W.H. (2010). Intranasal delivery to the central nervous system: Mechanisms and experimental considerations. J. Pharm. Sci..

[35] Duchêne D., Ponchel G., Wouessidjewe D. (1999). Cyclodextrins in targeting: Application to nanoparticles. Adv. Drug Deliv. Rev..

[36] Tiwari G., Tiwari R., Rai A.K. (2010). Cyclodextrins in delivery systems: Applications. J. Pharm. Bioallied Sci..

[37] Soares A.F., Carvalho Rde A., Veiga F. (2007). Oral administration of peptides and proteins: Nanoparticles and cyclodextrins as biocompatible delivery systems. Nanomedicine.

[38] Banks W.A., Engelke K., Hansen K.M., Bullock K.M., Calias P. (2019). Modest Blood-Brain Barrier Permeability of the Cyclodextrin Kleptose: Modification by Efflux and Luminal Surface Binding. J. Pharmacol. Exp. Ther..

[39] Zhong W., Xu L., Wang Q., Shen X. (2025). Formation of bovine serum albumin-galangin nanoparticles and their potential to inhibit reactive oxygen species-induced inflammation: Ethanol desolvation versus pH-shifting method. J. Dairy Sci..

[40] Bailus B.J., Scheeler S.M., Simons J., Sanchez M.A., Tshilenge K.T., Creus-Muncunill J., Naphade S., Lopez-Ramirez A., Zhang N., Lakshika Madushani K. (2021). Modulating FKBP5/FKBP51 and autophagy lowers HTT (huntingtin) levels. Autophagy.

[41] Mowafy H.A., Alanazi F.K., El Maghraby G.M. (2012). Development and validation of an HPLC–UV method for the quantification of carbamazepine in rabbit plasma. Saudi Pharm. J..

[42] Yang F., Dong Q., Chen Z., Gao B., Zheng D., Wang R., Qin S., Peng F., Luo M., Yang J. (2024). A pH-Responsive Drug-Delivery System Based on Apatinib-Loaded Metal-Organic Frameworks for Ferroptosis-Targeted Synergistic Anti-Tumor Therapy. Int. J. Nanomed..

[43] Soliman L., Party P., Nagy A., Farkas Á., Paróczai D., Burián K., Ambrus R. (2025). Enhanced pulmonary delivery of spray-dried theophylline: Investigation of the trehalose and amino acid combinations as innovative fine carriers. Eur. J. Pharm. Sci..

[44] Keller L.A., Merkel O., Popp A. (2022). Intranasal drug delivery: Opportunities and toxicologic challenges during drug development. Drug Deliv. Transl. Res..

[45] Sipos B., Szabó-Révész P., Csóka I., Pallagi E., Dobó D.G., Bélteky P., Kónya Z., Deák Á., Janovák L., Katona G. (2020). Quality by Design Based Formulation Study of Meloxicam-Loaded Polymeric Micelles for Intranasal Administration. Pharmaceutics.

[46] Avdeef A. (2003). Permeability. Absorption and Drug Development.

[47] Vincze A., Dékány G., Bicsak R., Formanek A., Moreau Y., Koplányi G., Takács G., Katona G., Balogh-Weiser D., Arany Á. (2023). Natural Lipid Extracts as an Artificial Membrane for Drug Permeability Assay: In Vitro and In Silico Characterization. Pharmaceutics.

[48] Rahimnejad M., Najafpour G., Bakeri G. (2012). Investigation and modeling effective parameters influencing the size of BSA protein nanoparticles as colloidal carrier. Colloids Surf. A Physicochem. Eng. Asp..

[49] Radwan S.E., El-Kamel A., Zaki E.I., Burgalassi S., Zucchetti E., El-Moslemany R.M. (2021). Hyaluronic-Coated Albumin Nanoparticles for the Non-Invasive Delivery of Apatinib in Diabetic Retinopathy. Int. J. Nanomed..

[50] Paik S.-Y.-R., Nguyen H.H., Ryu J., Che J.-H., Kang T.S., Lee J.K., Song C.W., Ko S. (2013). Robust size control of bovine serum albumin (BSA) nanoparticles by intermittent addition of a desolvating agent and the particle formation mechanism. Food Chem..

[51] Joseph D.J., Kathiresan V., Wilson B., Savitha B.K., Suma R. (2012). Design and Characterization of Bovine Serum Albumin Nanocarriers For Tenofovir by Modified Desolvation Method. J. Pharm. Res..

[52] (2025). Carbodiimide Crosslinker Chemistry. Protein Biology Methods Resource Library.

[53] Patra J.K., Das G., Fraceto L.F., Campos E.V.R., Rodriguez-Torres M.d.P., Acosta-Torres L.S., Diaz-Torres L.A., Grillo R., Swamy M.K., Sharma S. (2018). Nano based drug delivery systems: Recent developments and future prospects. J. Nanobiotechnol..

[54] Sanaeifar N., Mäder K., Hinderberger D. (2022). Macro- and Nanoscale Effect of Ethanol on Bovine Serum Albumin Gelation and Naproxen Release. Int. J. Mol. Sci..

[55] Danhier F., Feron O., Préat V. (2010). To exploit the tumor microenvironment: Passive and active tumor targeting of nanocarriers for anti-cancer drug delivery. J. Control. Release.

[56] Erukula S.V., Yochana S., Chatterjee P. (2016). Factors influencing the fabrication of albumin-bound drug nanoparticles (ABDns): Part II. Albumin-bound carbamazepine nanoparticles (ABCns). J. Microencapsul..

[57] Łagiewka J., Girek T., Ciesielski W. (2021). Cyclodextrins-Peptides/Proteins Conjugates: Synthesis, Properties and Applications. Polymers.

[58] Mardikasari S.A., Katona G., Sipos B., Ambrus R., Csóka I. (2023). Preparation and Optimization of Bovine Serum Albumin Nanoparticles as a Promising Gelling System for Enhanced Nasal Drug Administration. Gels.

[59] Zhang Y., Fu H., Liu D.E., An J., Gao H. (2019). Construction of biocompatible bovine serum albumin nanoparticles composed of nano graphene oxide and AIEgen for dual-mode phototherapy bacteriostatic and bacterial tracking. J. Nanobiotechnol..

[60] Scientific T. (2018). Bioconjugation Technical Handbook.

[61] Shang L., Wang Y., Jiang J., Dong S. (2007). pH-Dependent Protein Conformational Changes in Albumin:Gold Nanoparticle Bioconjugates:  A Spectroscopic Study. Langmuir.

[62] Akhtar S., AlAnsari R., Hasan B., Hasan S., Zayer A., AlHaddad J., Ansari M.A., Khan F.A., Ul-Hamid A., Henari F.Z. (2024). Anticancer and antibacterial potential of green synthesized BSA conjugated silver nanoparticles. J. Saudi Chem. Soc..

[63] Musuc A.M., Anuta V., Atkinson I., Sarbu I., Popa V.T., Munteanu C., Mircioiu C., Ozon E.A., Nitulescu G.M., Mitu M.A. (2021). Formulation of Chewable Tablets Containing Carbamazepine-?-cyclodextrin Inclusion Complex and F-Melt Disintegration Excipient. The Mathematical Modeling of the Release Kinetics of Carbamazepine. Pharmaceutics.

[64] Jain A.S., Date A.A., Pissurlenkar R.R.S., Coutinho E.C., Nagarsenker M.S. (2011). Sulfobutyl Ether7 β-Cyclodextrin (SBE7 β-CD) Carbamazepine Complex: Preparation, Characterization, Molecular Modeling, and Evaluation of In Vivo Anti-epileptic Activity. AAPS PharmSciTech.

[65] Kalanur S.S., Seetharamappa J., Kalalbandi V.K.A. (2010). Characterization of interaction and the effect of carbamazepine on the structure of human serum albumin. J. Pharm. Biomed. Anal..

[66] Loftsson T., Brewster M.E. (2010). Pharmaceutical applications of cyclodextrins: Basic science and product development. J. Pharm. Pharmacol..

[67] Smart J.D. (2005). The basics and underlying mechanisms of mucoadhesion. Adv. Drug Deliv. Rev..

[68] Carvalho F.C., Bruschi M.L., Evangelista R.C., Gremião M.P.D. (2010). Mucoadhesive drug delivery systems. Braz. J. Pharm. Sci..

[69] Smith J.S., MacRae R.J., Snowden M.J. (2005). Effect of SBE7-β-cyclodextrin complexation on carbamazepine release from sustained release beads. Eur. J. Pharm. Biopharm..

[70] Volkova T., Simonova O., Perlovich G. (2024). Mechanistic Insight in Permeability through Different Membranes in the Presence of Pharmaceutical Excipients: A Case of Model Hydrophobic Carbamazepine. Pharmaceutics.

[71] Baek E.J., Nguyen H.D., Ngo H.V., Gil M.C., Lee B.J. (2025). Long-term controlled release with reduced initial burst release utilizing calcium ion-triggering nanoaggregates of pasireotide-loaded fattigated albumin nanoparticles. Int. J. Pharm..

[72] Avdeef A. (2012). Absorption and Drug Development: Solubility, Permeability, and Charge State.

