# Supplementary material for: Design and In Vitro Evaluation of Cyclodextrin-Functionalized Albumin Nanoparticles for Intranasal Carbamazepine Brain Delivery
Source: Pharmaceutics. 2026 Mar 6;18(3):331. doi: 10.3390/pharmaceutics18030331 (PMC13029541; doi:10.3390/pharmaceutics18030331)
Supplement: Supplementary file 1 [file pharmaceutics-18-00331-s001.zip › pharmaceutics-4138473-supplementary.pdf]

Supplementary Materials

Design and in vitro evaluation of cyclodextrin-functionalized albumin nanoparticles for intranasal carbamazepine brain delivery

Hanan Mohammad[1], Maher Darwish[2,3], Mária Budai-Szűcs[1], Maryana Salamah[1], Rita Ambrus[1], György Tibor Balogh[4,5,6], Gábor Katona[1*], Ildikó Csóka[1]

[1] Institute of Pharmaceutical Technology and Regulatory Affairs, Faculty of Pharmacy, University of Szeged, H-6720 Szeged, Hungary; hanan.adnan.mohammad@yahoo.com (H.M.); budai-szucs.maria@szte.hu (M.B.S.); salamah.maryana@szte.hu (M.S); ambrus.rita@szte.hu (R.A.), katona.gabor@szte.hu (G.K.); csoka.ildiko@szte.hu (I.C.)

[2] Department of Optics and Quantum Electronics, University of Szeged, Dóm sq. 9, 6720 Szeged, Hungary; darwish_maher@ymail.com (M.D.)

[3] Department of Pharmaceutical Chemistry and Drug Control, Faculty of Pharmacy, Wadi International University, Homs, Syria; darwish_maher@ymail.com (M.D.)

[4] Department of Pharmaceutical Chemistry, Semmelweis University, Hőgyes Endre Str. 9, H-1092 Budapest, Hungary; .); balogh.gyorgy.tibor@semmelweis.hu (G.T.B.)

[5] Center for Pharmacology and Drug Research & Development, Semmelweis University, Üllői Str. 26, H-1085 Budapest, Hungary; balogh.gyorgy.tibor@semmelweis.hu (G.T.B.)

[6] Department of Chemical and Environmental Process Engineering, Budapest University of Technology and Economics, Műegyetem quay 3, H-1111 Budapest, Hungary; balogh.gyorgy.tibor@semmelweis.hu (G.T.B.)

* Correspondence: katona.gabor@szte.hu; Tel.: +36-62-545-575

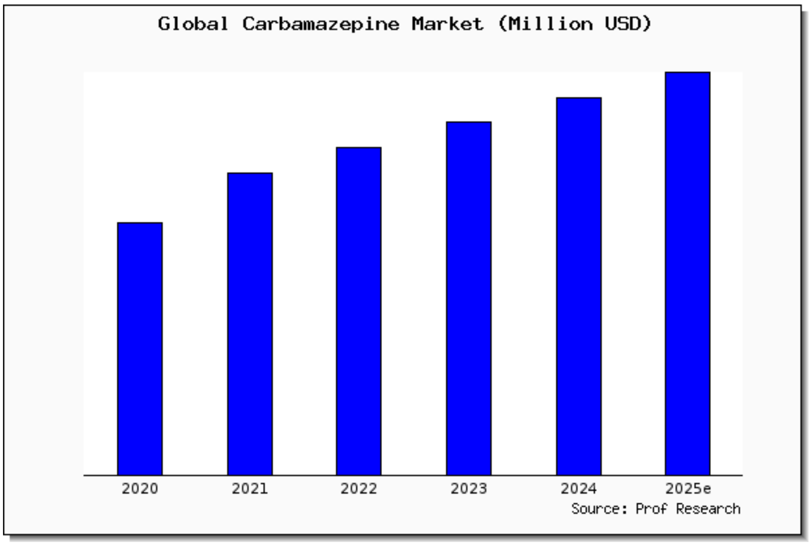

Figure S1. Carbamazepine Market Report about the global Carbamazepine market size in USD in 2025.

Table S1: The computer generated 18 experimental runs (factor 2 (B) CD type -1 means HβCD and +1 SβCD)

	A	B	C	D	E	F
Run	Factor 1<br>A: BSA concentration (mg/ml)	Factor 2<br>B: CD type	Factor 3<br>C: CD concentration (mg/ml)	Factor 4<br>D:PH	Factor 5<br>E: EtOH:BSA v/v	Factor 6<br>F: EDC:BSA %
**1**	10	-1	10	9	6	1
**2**	30	-1	1	9	6	5
**3**	10	1	10	9	1	5
**4**	10	1	1	9	6	1
**5**	30	-1	10	7	1	5
**6**	10	-1	1	9	1	5
**7**	10	-1	10	7	6	5
**8**	30	1	1	9	1	1
**9**	10	-1	1	7	1	1
**10**	20	-1	5.5	8	3.5	3
**11**	10	1	1	7	6	5
**12**	20	1	5.5	8	3.5	3
**13**	30	1	10	7	6	1
**14**	10	1	10	7	1	1
**15**	30	-1	1	7	6	1
**16**	30	1	10	9	6	5
**17**	30	1	1	7	1	5
**18**	30	-1	10	9	1	1

Table S2. DOE Response Surface Models

Response	Final Equation in Actual Factors
Particle Size (nm)	Size = -159.89 + 1.54×BSA - 122.34×CD type + 9.48×CD conc + 20.96×pH + 57.01×EtOH:BSA + 5.08×EDC:BSA + interactions: BSA×CD type (-4.42), CD type×pH (+25.86), etc.
PDI	PDI = -0.876 + 0.043×BSA + 0.306×CD type - 0.004×CD conc + 0.170×pH + 0.027×EtOH:BSA - 0.024×EDC:BSA + BSA×CD type (-0.007), BSA×pH (-0.006), CD type×EDC (-0.044)
Zeta Potential (mV)	Zeta = -58.37 + 3.06×BSA - 58.18×CD type + 0.70×CD conc + 3.39×pH + 0.41×EtOH:BSA + 0.32×EDC:BSA + BSA×CD type (+3.13), CD type×pH (+6.86), BSA×CD type×pH (-0.38)
Yield (%)	Yield = 60.30 + 1.16×BSA + 4.63×CD type + 0.20×CD conc - 2.28×pH + 1.79×EtOH:BSA + 1.10×EDC:BSA

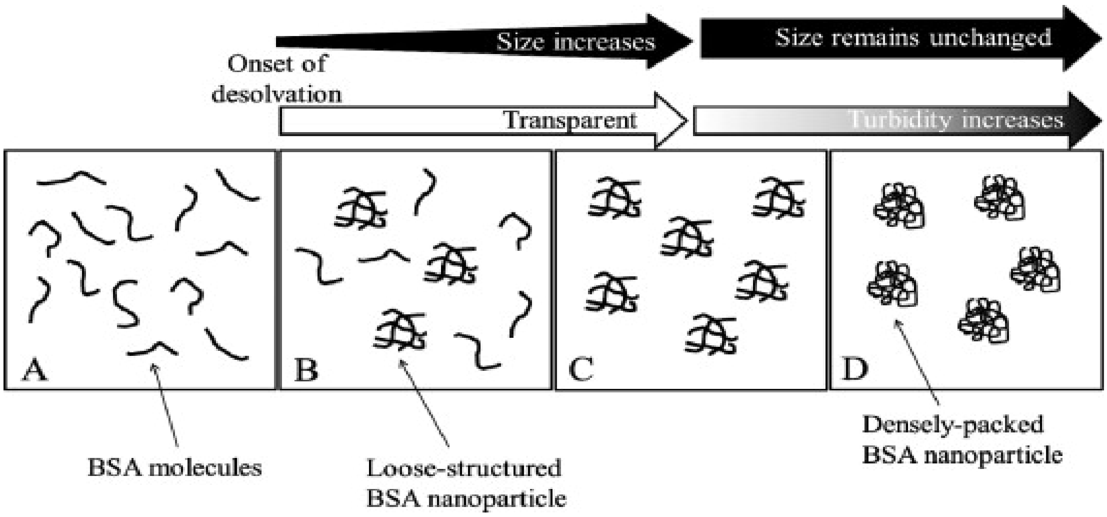

Figure S2: Illustration of the formation mechanism of BSA NPs by the desolvation process.

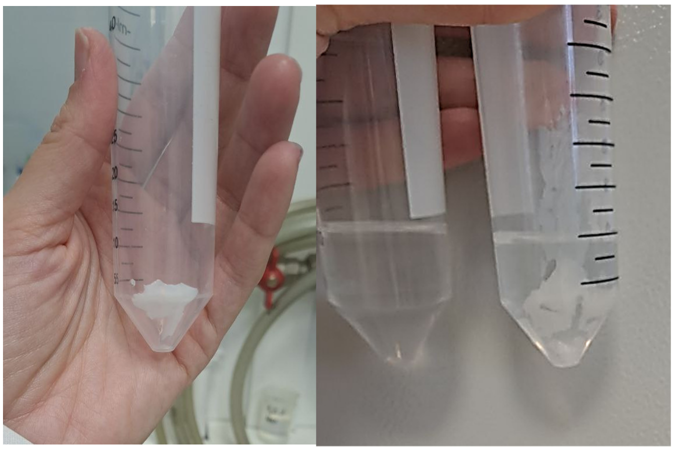

Figure S3. The qualitative gel residue on the edge of the tube after centrifuging.

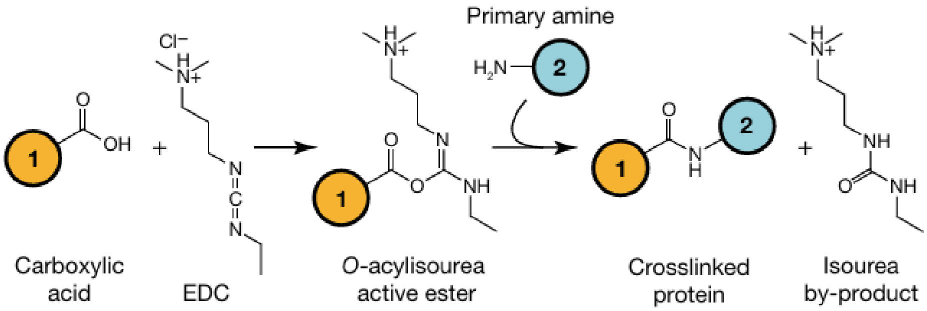

Figure S4. The mechanism of EDC as cross linker.

Table S3. Statistical comparison of physicochemical properties between CBZ@BSA NPs and CBZ@SβCD-BSA NPs.

Parameter	CBZ@BSA NPs (Mean ± SD)	CBZ@SβCD-BSA NPs (Mean ± SD)	t-statistic	df	p-value	Significant (α = 0.05)
Particle Size (nm)	145.17 ± 34.50	133.50 ± 16.26	0.530	2.85	0.635	No
PDI	0.34 ± 0.04	0.26 ± 0.07	1.746	3.12	0.176	No
DL (%)	31.91 ± 0.01	34.28 ± 0.43	-9.609	2.00	0.011*	Yes
EE (%)	38.10 ± 0.01	41.02 ± 0.51	-9.895	2.00	0.010*	Yes

* Statistically significant at $p < 0.05$.

Table S4. Similarity factor (f2) for the *in vitro* release profiles of CBZ formulations.

Comparison	Medium	No. of Time Points	f2 Value	Conclusion
CBZ-BSA NPs vs. CBZ-SβCD-BSA NPs	PBS (pH 6.8)	7	69.74	Similar
Free CBZ vs. CBZ-BSA NPs	PBS (pH 6.8)	7	44.17	Dissimilar
Free CBZ vs. CBZ-SβCD-BSA NPs	PBS (pH 6.8)	7	40.84	Dissimilar
